# Non‐Coding Transcripts From Diversified Members of IgLec Family Protect Antiviral Effectors From Viral miRNA

**DOI:** 10.1002/advs.77981

**Published:** 2026-09-27

**Authors:** Ying Huang, Rui Shen, Xin Huang, Gu‐Qin Ji, Jiang‐Feng Lan, Qian Ren

**Affiliations:** ^1^ Jiangsu Province Engineering Research Center For Marine Bio‐Resources Sustainable Utilization, College of Oceanography Hohai University Nanjing China; ^2^ State Key Laboratory of Climate System Prediction and Risk Management, School of Marine Sciences Nanjing University of Information Science and Technology Nanjing China; ^3^ Jiangsu Province Engineering Research Center For Aquatic Animals Breeding and Green Efficient Aquacultural Technology, College of Marine Science and Engineering Nanjing Normal University Nanjing China; ^4^ College of Veterinary Medicine, Shandong Provincial Key Laboratory of Zoonoses Shandong Agricultural University Taian China

**Keywords:** genomic diversification, host‐pathogen interaction, intron retention, multi‑locus duplication, viral miRNA decoy

## Abstract

Immune gene families frequently produce numerous non‐coding transcripts, yet their biological functions remain largely unexplored. Here, using the arthropod *immunoglobulin domain‐containing lectin* (*IgLec*) family as a paradigm, we provide evidence that this complexity may reflect a critical defensive function for non‐coding transcripts in antiviral immunity. *IgLec* is restricted to decapod infraorders (Brachyura and Astacidea), and a structurally related bacterial homolog suggests its origin may involve horizontal gene transfer. The *IgLec* family generates extraordinary transcript diversity through three mechanisms, multi‐locus duplication, inter‐locus recombination, and extensive alternative splicing, yielding both protein‐coding and non‐coding transcripts. Protein‐coding IgLec variants restrict viral replication by inducing *antimicrobial peptide* expression. Upon white spot syndrome virus infection, non‐coding transcripts (e.g., intron‐retaining transcripts) are preferentially targeted by virus‐encoded microRNA‐N48, thereby protecting protein‐coding isoforms from repression. Loss of these decoy transcripts compromises antiviral defense and increases host mortality. Collectively, these results reveal a decoy‐based antiviral strategy in which non‐coding transcripts safeguard immune effectors from pathogen subversion, uncovering an unrecognized layer of innate immunity.

## Graphical abstract

The IgLec gene family generates both protein‐coding antiviral effectors and non‐coding transcripts. Upon viral infection, non‐coding transcripts are preferentially targeted by viral miR‐N48, thereby buffering protein‐coding isoforms from repression. Depletion of these decoy transcripts compromises antiviral defense, revealing a non‐coding RNA‐mediated protective layer in innate immunity.

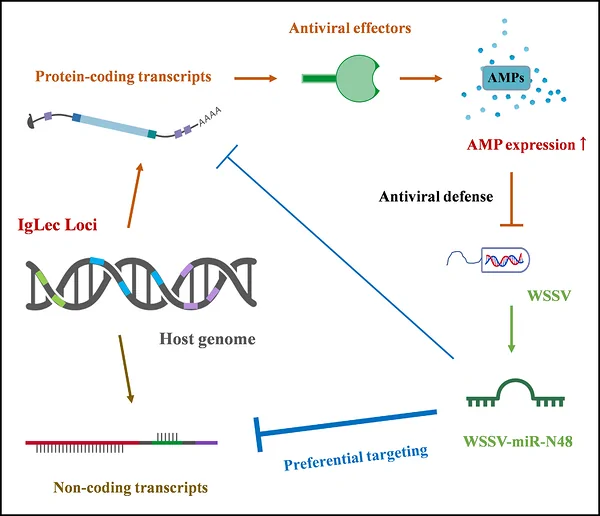

## 1 Introduction

For decades, the study of immune gene diversification has been anchored in a protein‐centric dogma: genomic complexity evolves primarily to expand the repertoire of receptors capable of recognizing a universe of pathogens [1, 2]. This paradigm, rooted in the success of adaptive immunity and the expansion of innate immune gene families, posits that the selective advantage lies in the functional diversity of protein products [3]. Consequently, the pervasive generation of non‐coding transcripts from these loci, particularly those arising from alternative splicing and retained introns, has been largely relegated to transcriptional noise or inconsequential byproducts of a noisy genome [4, 5, 6].

This view is particularly entrenched in arthropod immunity [7, 8]. Devoid of adaptive somatic recombination, invertebrates like crustaceans are textbook examples of this protein‐centric logic, relying on the germline‐encoded diversity of pattern recognition receptors (PRRs), such as C‐type lectins, to detect infection [9, 10]. Among these, the immunoglobulin domain‐containing lectin (IgLec) family, a lectin subclass distinguished by an immunoglobulin (IG) domain juxtaposed with a canonical carbohydrate recognition domain (CRD), has been identified in several decapod crustaceans, though its functional significance has remained enigmatic [11, 12]. The prevailing assumption has been that, like other PRRs, its function is encoded in its protein sequence.

However, a quiet revolution in our understanding of gene regulation now compels us to reexamine this assumption. Non‐coding transcripts, including those derived from intron retention, can act as dynamic post‐transcriptional regulators, functioning as decoy transcripts or molecular sponges [13, 14]. Notably, immune gene families in diverse organisms have been increasingly recognized to generate a complex landscape of alternatively spliced transcripts, many of which are non‐coding transcripts, yet their functional relevance remains largely unexplored. This raises a fundamental and largely unexplored question in evolutionary immunology. Could the seemingly wasteful transcriptional complexity of innate immune genes be co‐opted as a regulatory defense mechanism in the host‐pathogen arms race? If confirmed, this would challenge the traditional view of transcriptional diversity in immune genes as largely incidental. This question assumes urgency given that viruses, including the devastating white spot syndrome virus (WSSV), encode their own microRNAs (miRNAs) to subvert host defenses [15]. WSSV‐encoded miRNAs have been shown to target host immune genes, facilitating viral immune evasion [16, 17]. Yet whether host‐derived non‐coding transcripts participate directly in such conflicts remains unknown.

Here, we address this question by systematically characterizing the *IgLec* gene family in the Chinese mitten crab, *Eriocheir sinensis*, a natural host for WSSV. We uncover an unexpected regulatory logic. Through multi‐locus duplication, inter‐locus recombination, and extensive alternative splicing, the *IgLec* family generates both protein‐coding effectors and a diverse suite of non‐coding transcripts. Among these non‐coding transcripts, we selected intron‐retaining transcripts as representative candidates for functional investigation. These intron‐retaining non‐coding transcripts are preferentially targeted by WSSV‐encoded miR‐N48, thereby buffering the viral attack and shielding their protein‐coding counterparts from suppression. Our findings suggest that genomic complexity can be co‐opted to build a post‐transcriptional buffering system against viral subversion, providing a new perspective for understanding immune gene evolution.

## 2 Results

### 2.1 Phylogenetic Distribution, Domain Architecture, and Sequence Conservation of IgLec

**FIGURE 1:**
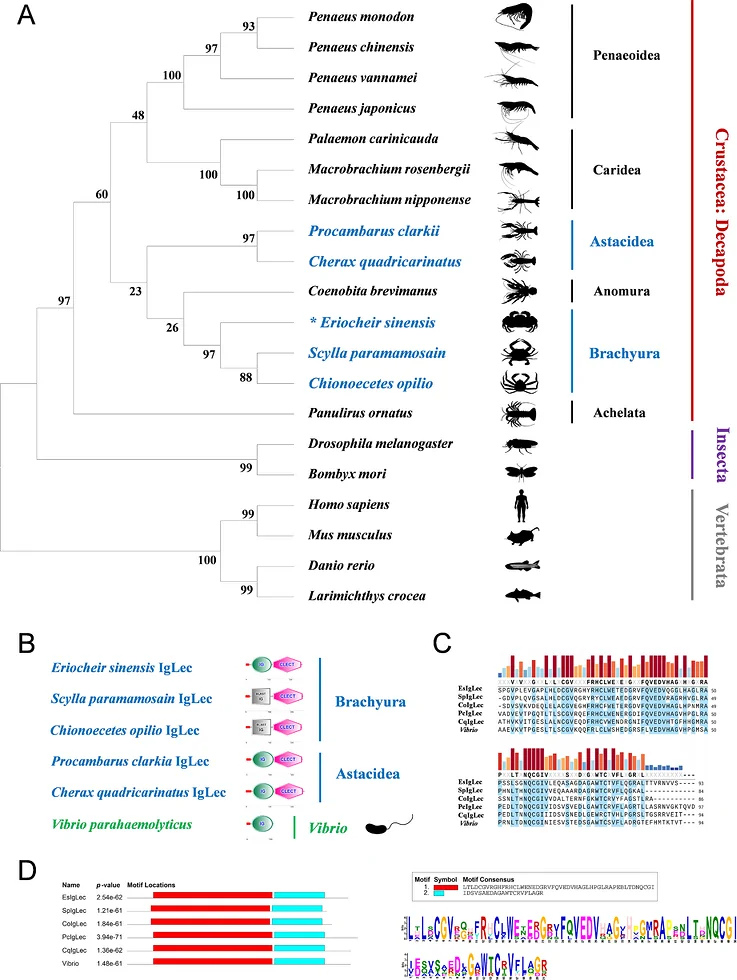
Phylogenetic and comparative analysis of IgLec. (A) Phylogenetic tree constructed based on *cox1* gene from 20 species using MEGA 11. The five species in which IgLec orthologs were identified, belonging to Brachyura and Astacidea, are highlighted in blue. (B) Domain architecture of IgLec proteins from the five crustacean species and *V. parahaemolyticus* predicted by SMART. (C) Multiple sequence alignment of the IG domain from the five crustacean species and *V. parahaemolyticus*. Identical residues (> 80% identity) are shaded in blue. (D) Conserved motifs within the IG domain identified by MEME analysis.

A phylogenetic tree was constructed based on *mitochondrial cytochrome c oxidase subunit I* (*cox1*) genes from 20 species to illustrate the taxonomic relationships among the examined taxa. Genomic, transcriptomic, and database searches revealed that IgLec orthologs are present exclusively in five decapod species within Brachyura and Astacidea, including *E. sinensis*, *Scylla paramamosain*, *Chionoecetes opilio*, *Procambarus clarkii*, and *Cherax quadricarinatus*, whereas no homologs were detected in other arthropod lineages (Figure 1A). All five crustacean IgLec proteins share a tripartite architecture consisting of an N‐terminal signal peptide, an IG domain, and a CRD. A homologous protein identified in the bacterium *Vibrio parahaemolyticus* contains a signal peptide and IG domain but lacks the CRD, raising the possibility of horizontal gene transfer from bacteria to decapod crustaceans (Figure 1B). Multiple sequence alignment revealed striking conservation of the IG domain across the five crustacean species and *V. parahaemolyticus* (Figure 1C). Conserved motifs identified by MEME analysis, ranging from 21 to 50 amino acids in length, further support functional constraint acting on this domain across kingdoms (Figure 1D).

### 2.2 Genomic Architecture Generates Diverse Coding and Non‐Coding Transcripts Within the IgLec Gene Family

**FIGURE 2:**
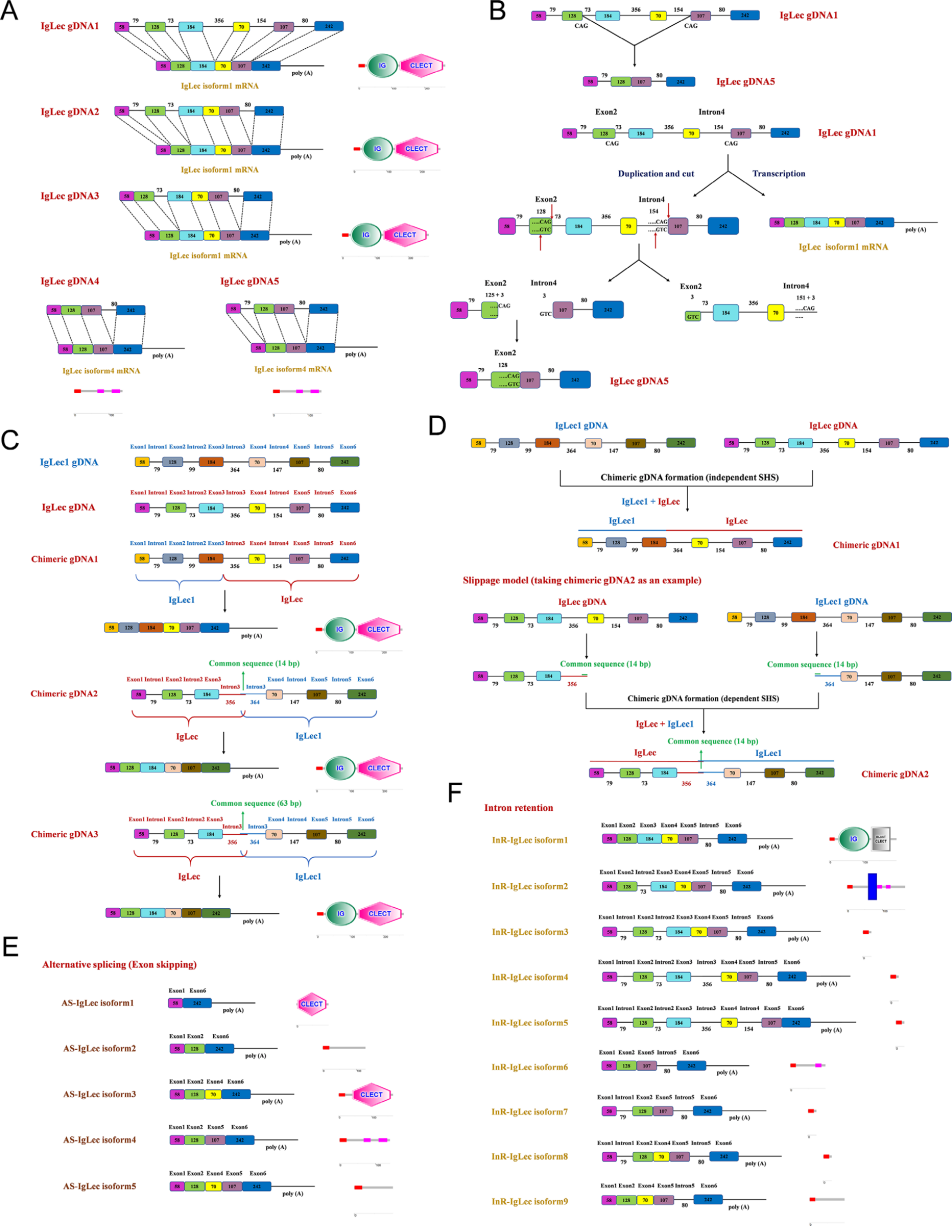
Genomic structures and rearrangement mechanisms of *IgLec* loci and its alternatively spliced isoforms. (A) Structural organization of five homologous *IgLec* genomic loci (*IgLec gDNA1‐5*). *gDNA1‐3* each contain six exons and encode full‐length *IgLec isoform1*, whereas *gDNA4‐5* each contain four exons and encode a truncated *IgLec isoform4*. Note the differential intron composition among loci. (B) Proposed origin of *gDNA5* from *gDNA1* via non‐allelic homologous recombination mediated by direct repeat sequences (CAG) located at the end of exon 2 and the terminus of intron 4, resulting in precise deletion of exons 3 and 4. (C) Schematic structures of three chimeric genes (*Chimeric gDNA1‐3*) generated by inter‐locus recombination between *IgLec* and *IgLec1*. (D) Formation mechanisms of chimeric genes. *Chimeric gDNA1* arose through non‐canonical recombination independent of short homologous sequences, whereas *Chimeric gDNA2‐* were formed via homologous recombination mediated by short direct repeats of 14 and 63 bp within intron 3. (E) Five exon‐skipping splice variants (*AS‐IgLec isoform1‐5*). (F) Nine intron‐retention transcripts (*InR‐IgLec isoform1‐9*), generated by retention of individual introns or combinations thereof.

To elucidate the genomic basis of structural diversity in the *IgLec* gene family, the genomic organization of *IgLec* loci in *E. sinensis* was characterized. PCR amplification and sequencing identified five homologous genomic loci (*IgLec gDNA1‐5*) (Figure 2A). Among these, *gDNA1‐3* each contain six exons and encode the full‐length *IgLec isoform1*, which consists of a signal peptide, an IG domain, and a CRD. These full‐length loci differ in intron composition, *gDNA1* contains all five introns, *gDNA2* lacks introns 3 and 4, and *gDNA3* retains only introns 2 and 5. By contrast, *gDNA4* and *gDNA5* each contain only four exons (exons 1, 2, 5, and 6) and encode a truncated isoform (*IgLec isoform4*) lacking specific structural domains; these truncated loci also differ in intron composition, with *gDNA4* retaining intron 5 only and *gDNA5* retaining introns 1 and 5 (Figure 2A). Comparative genomic analysis revealed that *gDNA5* likely originated from *gDNA1* through a non‐allelic homologous recombination event, retaining exons 1, 2, 5, and 6 but exhibiting precise deletion of exons 3 and 4 along with the intervening intronic sequence (Figure 2B). Sequence alignment demonstrated that this deletion is mediated by direct repeat sequences (CAG) located at the end of exon 2 and the terminus of intron 4 (Figure 2B).

Further analysis uncovered inter‐locus recombination events between *IgLec* and a homologous gene, *IgLec1*, generating three chimeric genes (*Chimeric gDNA1‐3*) (Figure 2C). *IgLec* and *IgLec1* share identical exon‐intron organization, and all three chimeric genes are predicted to encode proteins retaining the full‐length domain architecture (signal peptide, IG domain, and CRD), albeit with distinct amino acid sequences. Mechanistic analysis revealed distinct formation pathways: *Chimeric gDNA1* arose through non‐canonical recombination independent of short homologous sequences, whereas *Chimeric gDNA2* and *gDNA3* were formed via homologous recombination mediated by short direct repeats of 14 and 63 bp within intron 3, likely through a replication slippage mechanism (Figure 2D).

Transcriptome analysis combined with cDNA cloning revealed extensive alternative splicing, yielding two major categories of splice variants. Five exon‐skipping isoforms (*AS‐IgLec isoform1‐5*) were identified (Figure 2E). Among these, *AS‐IgLec isoform3* lacks several exons but maintains an intact reading frame, producing a shortened protein that retains the CRD. In contrast, the other four exon‐skipping variants either lack critical exons encoding essential structural domains or contain disrupted reading frames and are predicted to produce truncated proteins. Together with the full‐length *IgLec isoform1*, *AS‐IgLec isoform3* served as representative protein‐coding variants for subsequent functional characterization. Additionally, nine intron‐retention transcripts (*InR‐IgLec isoform1‐9*) were identified, characterized by retention of individual introns or combinations thereof (Figure 2F). Sequence analysis indicated that most of these transcripts contain premature stop codons and are predicted to be non‐coding transcripts. Transcripts retaining intron 3 (*InR‐intron3*) or intron 5 (*InR‐intron5*) were selected as representative non‐coding transcripts for functional studies.

### 2.3 Alternative Splicing Generates Non‐Coding Transcripts Across Multiple IgLec Family Members

**FIGURE 3:**
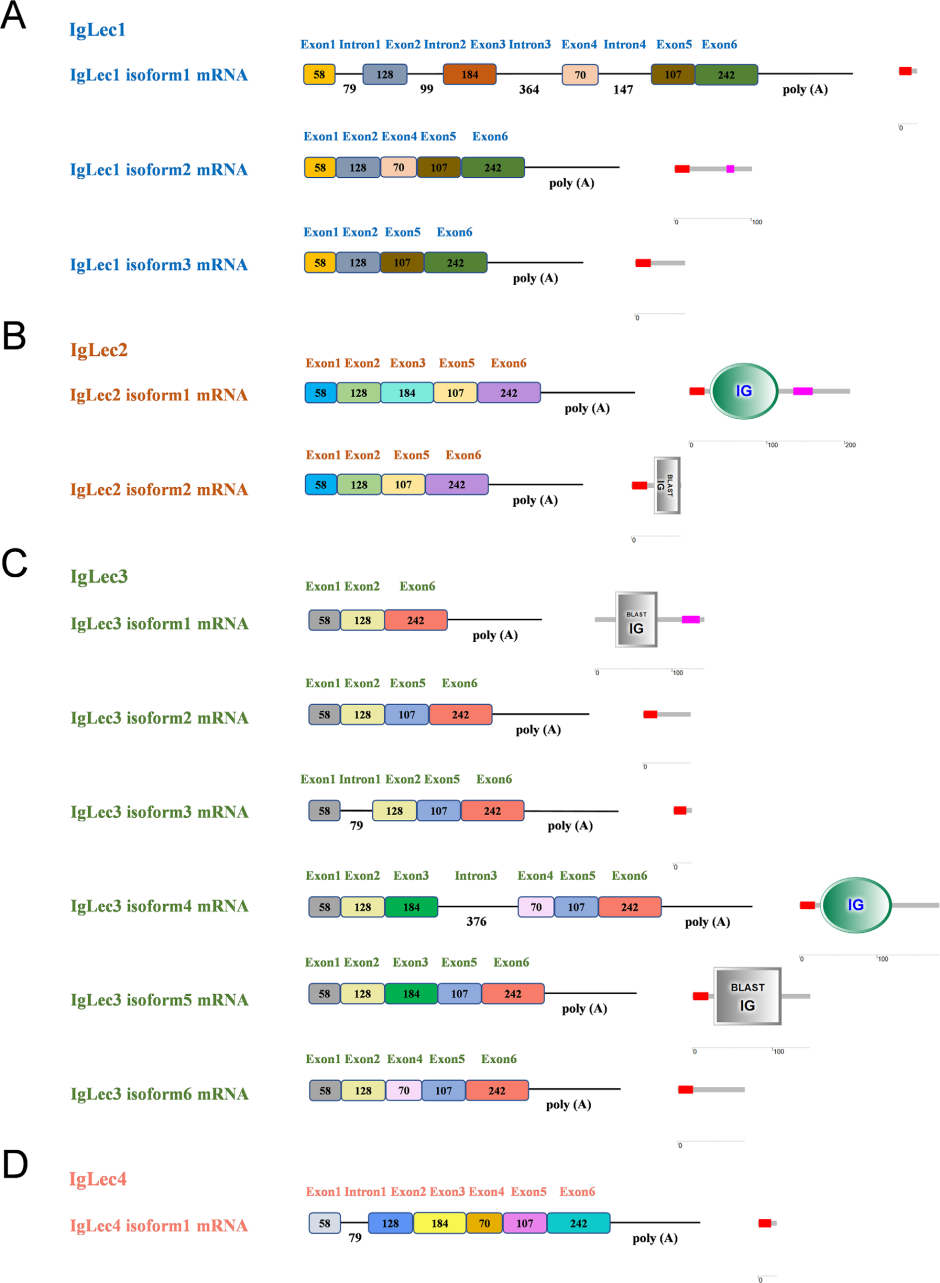
Identification of alternatively spliced isoforms in the *IgLec* gene family. Schematic representation of alternatively spliced isoforms identified in four family members: (A) *IgLec1* (*isoform1–3*), (B) *IgLec2* (*isoform1–2*), (C) *IgLec3* (*isoform1–6*), and (D) *IgLec4* (*isoform1*). Alternative splicing events, involving exon skipping, intron retention, or both, were observed across all four genes.

To determine whether the production of non‐coding transcripts via alternative splicing is specific to *IgLec* or represents a broader characteristic of this lectin family, the analysis was extended to four family members, *IgLec1* through *IgLec4*. Alternative splicing events, including exon skipping, intron retention, or both, were observed across all four genes. Specifically, *IgLec1* produced three splice variants (*isoform1–3*) (Figure 3A), *IgLec2* produced two variants (*isoform1–2*) (Figure 3B), *IgLec3* produced six variants (*isoform1–6*) (Figure 3C), and *IgLec4* produced one variant (*isoform1*) (Figure 3D). These results demonstrate that the generation of non‐coding transcripts via alternative splicing is not restricted to *IgLec* but is a shared feature across multiple members of this lectin family.

### 2.4 Protein‐Coding IgLec Isoforms Suppress WSSV Replication and Enhance Host Survival

**FIGURE 4:**
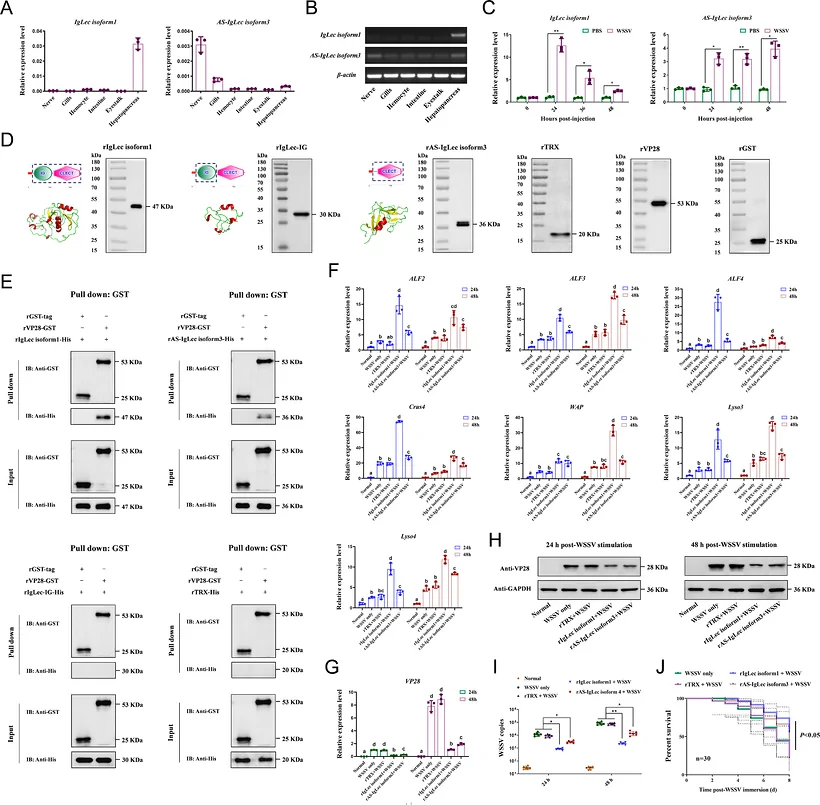
Expression profiles and functional characterization of protein‐coding IgLec isoforms. (A, B) Tissue‐specific expression of *IgLec isoform1* and *AS‐IgLec isoform3* analyzed by RT‐qPCR (A) and semiquantitative RT‐PCR (B), with *β‐actin* as reference. (C) Temporal expression patterns following WSSV infection at 0, 24, 36, 48 hpi. (D) Immunoblot analysis of purified recombinant proteins (rIgLec isoform1, rIgLec‑IG, and rAS‑IgLec isoform3). (E) GST pull‐down assay assessing the interaction of rIgLec isoform1, rIgLec‐IG, or rAS‐IgLec isoform3 with VP28. Immobilized rVP28‐GST was incubated with each His‐tagged recombinant protein (or rTRX as a negative control). Bound proteins were eluted and detected by Western blot using both anti‐His and anti‐GST antibodies. (F) Expression of *AMP* genes (*ALF2‐4*, *Crus4*, *WAP*, *Lyso3‐4*) at 24 and 48 hpi after injection of rIgLec isoforms pre‐incubated with WSSV. (G) Relative *VP28* transcript levels. (H) Western blot analysis of VP28 protein accumulation; GAPDH served as loading control. (I) WSSV genomic copy numbers. (J) Kaplan‐Meier survival curves. Data are mean ± SD (n = 3). Statistical significance was determined by Student's *t*‐test (C, J) or one‐way ANOVA with post‐hoc Tukey test (F, G). ^*^ *p* < 0.05, ^**^ *p* < 0.01; different letters indicate significant differences at *p* <0.05.

To investigate the functional roles of protein‐coding IgLec variants, the tissue distribution, expression patterns, and antiviral activities of IgLec isoform1 and AS‐IgLec isoform3 were characterized. Reverse transcription‐quantitative PCR (RT‐qPCR) revealed distinct tissue distribution patterns. *IgLec isoform1* was predominantly expressed in the hepatopancreas, whereas *AS‐IgLec isoform3* exhibited broader distribution with the highest expression in the nerve, followed by gills and hepatopancreas (Figure 4A,B). Following WSSV challenge, both isoforms responded to infection but with distinct patterns. *IgLec isoform1* expression was rapidly upregulated 12.64‐fold at 24 h post‐injection (hpi), then declined but remained above baseline. *AS‐IgLec isoform3* showed sustained upregulation, progressively increasing to a 3.96‐fold peak at 48 hpi (Figure 4C).

To directly assess antiviral functions, recombinant proteins were expressed and purified (Figure 4D). GST pull‑down assays were performed to identify the domain responsible for binding to the WSSV envelope protein VP28. rIgLec isoform1 and rAS‑IgLec isoform3 both bound to VP28, with the full‑length protein showing stronger interaction. In contrast, the isolated IG domain (rIgLec‑IG) did not bind VP28 (Figure 4E). Notably, AS‐IgLec isoform3 retains an intact CRD, consistent with its ability to bind VP28 and supporting the conclusion that the CRD mediates this interaction. When pre‐incubated with WSSV and injected into crabs, both rIgLec isoform1 and rAS‐IgLec isoform3 significantly upregulated multiple *antimicrobial peptide* (*AMP*) genes, including *anti‐lipopolysaccharide factor 2‐4* (*ALF2‐4*), *crustin 4* (*Crus4*), *whey acidic protein* (*WAP*), and *lysozyme 3‐4* (*Lyso3‐4*), at 24 and 48 hpi (Figure 4F). rIgLec isoform1 induced significantly higher *AMP* expression than rAS‐IgLec isoform3. Consistent with enhanced *AMP* expression, both recombinant proteins suppressed WSSV replication, as evidenced by reduced *VP28* transcript levels (Figure 4G), decreased VP28 protein accumulation (Figure 4H), and lower viral genomic copy numbers (Figure 4I). rIgLec isoform1 exhibited greater antiviral potency than rAS‐IgLec isoform3. Consequently, both isoforms conferred protection against WSSV‐induced mortality, with rIgLec isoform1 providing superior survival benefit (Figure 4J).

### 2.5 Intron‐Retaining Non‐Coding Transcripts Protect Protein‐Coding Isoforms During WSSV Infection

**FIGURE 5:**
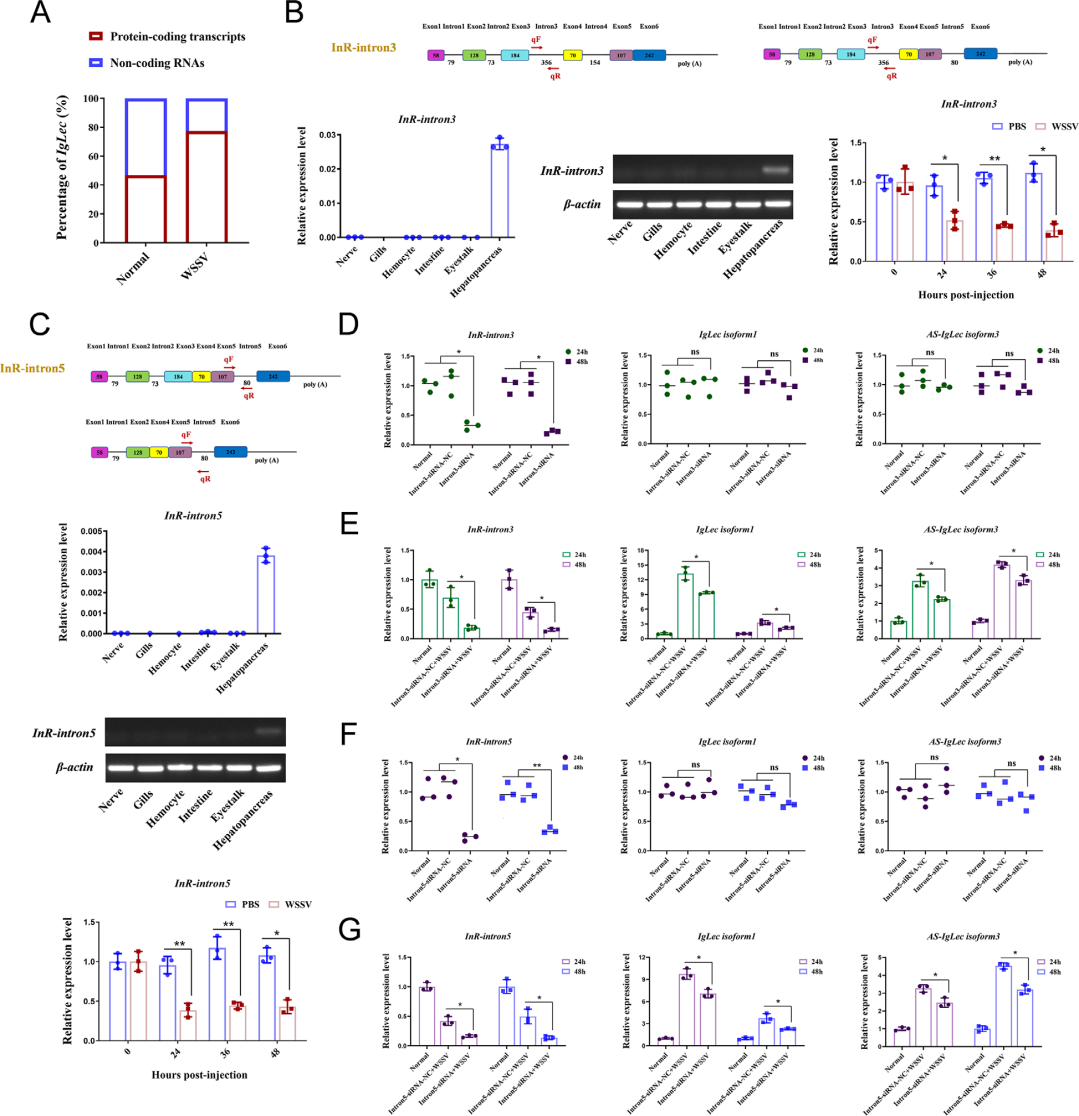
Expression profiles and functional assessment of intron‐retaining non‐coding *IgLec* transcripts. (A) Differential induction of protein‐coding *IgLec* transcripts and non‐coding *IgLec* transcripts in *E. sinensis* following WSSV challenge. Relative frequencies were determined by PCR cloning and sequencing. (B, C) Tissue‐specific distribution and temporal expression patterns of *InR‐intron3* (B) and *InR‐intron5* (C) upon WSSV infection. (D, E) Effects of *InR‐intron3* knockdown on the expression of *InR‐intron3*, *IgLec isoform1*, and *AS‐IgLec isoform3* under basal conditions (D) or after WSSV challenge (E). (F, G) Effects of *InR‐intron5* knockdown under basal conditions (F) or after WSSV challenge (G). Data are mean ± SD (n = 3). Student's *t*‐test, ^*^ *p* < 0.05, ^**^ *p* < 0.01; ns, not significant.

We next quantified the relative abundance of protein‐coding and non‐coding *IgLec* transcripts, with over 500 independent clones sequenced per group from hepatopancreas tissues collected at 24 h post‐WSSV infection (Figure 5A). Transcripts were classified based on open reading frame (ORF) prediction: those containing a complete ORF were designated as protein‐coding, whereas those with premature stop codons or disrupted ORFs were designated as non‐coding transcripts. In uninfected crabs, non‐coding transcripts dominated the total transcript pool, accounting for 53.2%, while protein‐coding isoforms constituted only 46.8%. Upon WSSV infection, this transcriptional ratio underwent a striking reversal, protein‐coding transcripts increased sharply to 77.4%, whereas non‐coding transcript abundance decreased markedly to 22.6%. Both *InR‐intron3* and *InR‐intron5* transcripts were predominantly expressed in the hepatopancreas across detected tissues (Figure 5B,C). Temporal expression analysis further revealed their significant downregulation during WSSV infection. Specifically, *InR‐intron3* levels decreased to 0.52‐fold at 24 hpi and 0.39‐fold at 48 hpi, while *InR‐intron5* abundance declined to 0.39‐fold at 24 hpi and 0.43‐fold at 48 hpi (Figure 5B,C). This rapid and persistent reduction in non‐coding transcript abundance during viral challenge strongly supports their active involvement in host antiviral responses.

To assess functional relevance, small interfering RNA (siRNA)‐mediated knockdown of these transcripts was performed. Under basal conditions, knockdown of either *InR‐intron3* (Figure 5D) or *InR‐intron5* (Figure 5F) transcripts did not affect expression of protein‐coding isoforms *IgLec isoform1* or *AS‐IgLec isoform3*. However, when knockdown was performed prior to WSSV challenge, both protein‐coding isoforms were significantly downregulated at 24 and 48 hpi (Figure 5E,G). These results indicate that intron‐retaining transcripts, while non‐coding themselves, are dispensable for maintaining basal expression but become essential for protecting protein‐coding isoforms specifically during viral infection.

### 2.6 WSSV‐Encoded miR‐N48 Targets IgLec and Preferentially Downregulates Non‐Coding Transcripts

**FIGURE 6:**
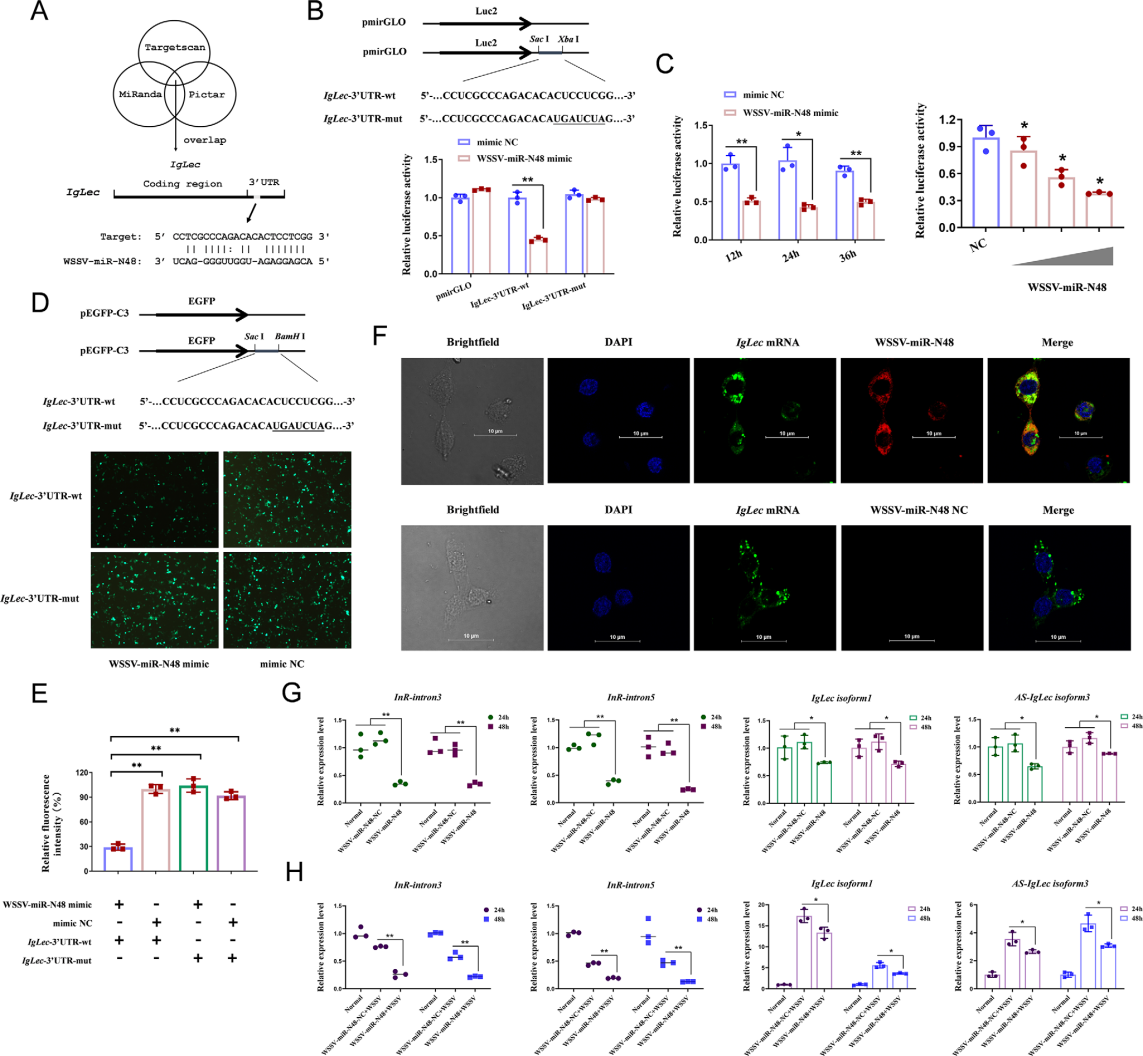
Validation of *IgLec* as a direct target of WSSV‐encoded miR‐N48. (A) Predicted WSSV‐miR‐N48 binding site within *IgLec* 3’UTR. (B) Luciferase reporter assay validating direct targeting. (C) Dose‐ and time‐dependent repression of luciferase activity by WSSV‐miR‐N48. (D, E) EGFP‐based reporter assay confirming targeting specificity. Representative fluorescence images (D) and quantitative GFP intensity (E). (F) The co‐localization of WSSV‐miR‐N48 and *IgLec* mRNA in crab hemocytes, WSSV‐miR‐N48, *IgLec* mRNA, and nucleus of hemocytes were respectively detected with FAM‐labeled *IgLec* mRNA probe (green), Cy3‐labeled WSSV‐miR‐N48 probe (red) and DAPI (blue). (G, H) In vivo regulation of *IgLec* transcripts by WSSV‐miR‐N48 agomir under basal conditions (G) or following WSSV challenge (H). Data are mean ± SD (n = 3). Student's *t*‐test, ^*^ *p* < 0.05, ^**^ *p* < 0.01.

Bioinformatic prediction identified a putative WSSV‐miR‐N48 binding site within the *IgLec* 3’ untranslated region (UTR) (Figure 6A). Luciferase reporter assays validated this interaction, showing significant repression of the wild‑type reporter but not the mutant (Figure 6B). The repression was dose‐dependent and sustained through 36 h post‐transfection (hpt) (Figure 6C). EGFP‐based reporter assays further confirmed direct targeting, co‐transfection of the wild‐type *IgLec* 3’UTR with WSSV‐miR‐N48 mimic reduced GFP fluorescence to 29.2% of the control value, whereas mutant 3’UTR was unaffected (Figure 6D,E). To examine subcellular localization, we performed fluorescence in situ hybridization (FISH) on crab hemocytes. Confocal microscopy revealed co‑localization of WSSV‑miR‑N48 and *IgLec* mRNA in these cells (Figure 6F).

In vivo, injection of WSSV‐miR‐N48 agomir significantly reduced expression of all *IgLec* transcripts in crab hepatopancreas (Figure 6G,H). The non‐coding intron‐retaining transcripts exhibited greater sensitivity to miRNA‐mediated repression than the protein‐coding isoforms under both basal conditions and WSSV challenge. *InR‐intron3* and *InR‐intron5* transcripts were reduced to 0.2‐ to 0.4‐fold of control levels, whereas *IgLec isoform1* and *AS‐IgLec isoform3* were reduced to only 0.6‐ to 0.9‐fold (Figure 6G,H). This preferential downregulation suggests that these non‐coding transcripts may serve as decoy transcripts that buffer protein‐coding isoforms from viral miRNA‐mediated suppression.

### 2.7 Intron‐Retaining Transcripts Protect Protein‐Coding Isoforms From WSSV‐miR‐N48‐Mediated Suppression

**FIGURE 7:**
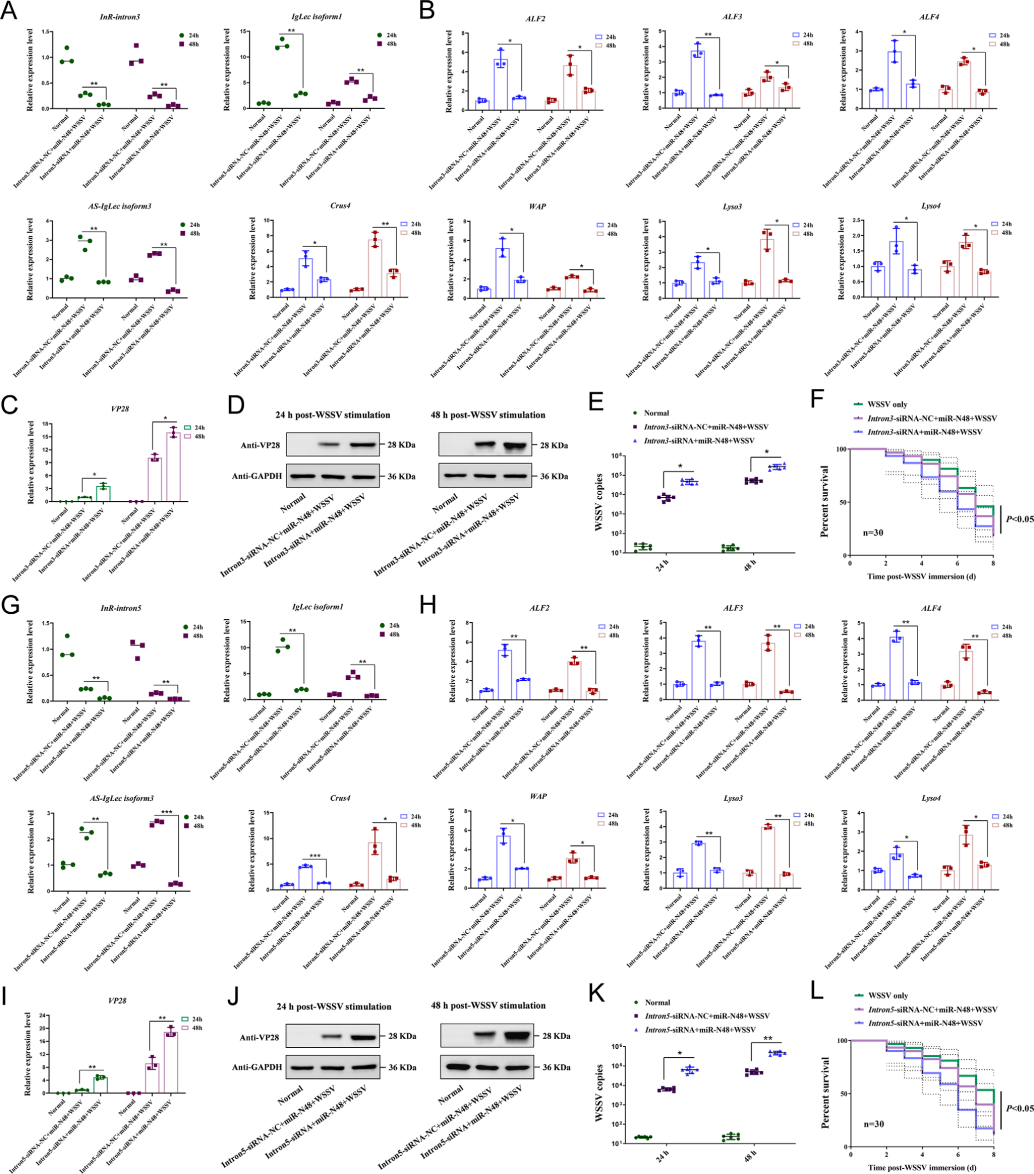
Functional interplay between intron‐retaining transcripts and WSSV‐miR‐N48 in regulating protein‐coding IgLec isoforms. (A‐F) Experiments following *InR‐intron3* knockdown. (A) Expression of *InR‐intron3*, *IgLec isoform1*, and *AS‐IgLec isoform3* after knockdown and subsequent challenge with WSSV‐miR‐N48 agomir + WSSV. (B) Expression of *AMP* genes. (C) *VP28* transcript levels. (D) VP28 protein accumulation. (E) WSSV genomic copy numbers. (F) Cumulative survival rates. (G‐L) Experiments following *InR‐intron5* knockdown. (G) Expression of *InR‐intron5*, *IgLec isoform1*, and *AS‐IgLec isoform3* after knockdown and subsequent challenge with WSSV‐miR‐N48 agomir + WSSV. (H) Expression of *AMP* genes. (I) *VP28* transcript levels. (J) VP28 protein accumulation. (K) WSSV genomic copy numbers. (L) Cumulative survival rates. Data are mean ± SD (n = 3). Student's *t*‐test, ^*^ *p* <0.05, ^**^ *p* < 0.01, ^***^ *p* < 0.001.

The preferential downregulation of intron‐retaining transcripts by WSSV‐miR‐N48 prompted an investigation of whether these non‐coding transcripts protect protein‐coding isoforms from viral miRNA attack. *InR‐intron3* or *InR‐intron5* transcripts were knocked down prior to co‐injection with WSSV‐miR‐N48 agomir and WSSV. When *InR‐intron3* transcripts were depleted, subsequent exposure to WSSV‐miR‐N48 and WSSV resulted in significantly reduced expression of both *IgLec isoform1* and *AS‐IgLec isoform3* at 24 and 48 hpi (Figure 7A). This loss of protein‐coding isoform expression was accompanied by downregulation of multiple *AMP* genes (Figure 7B), increased *VP28* transcript levels (Figure 7C), elevated VP28 protein accumulation (Figure 7D), and higher WSSV genomic copy numbers (Figure 7E). Consequently, survival rates were significantly reduced in crabs lacking *InR‐intron3* transcripts prior to viral challenge (Figure 7F). Thus, depletion of *InR‑intron3* led to reduced protein‐coding isoform expression, impaired *AMP* induction, increased WSSV replication, and decreased host survival; virtually identical results were obtained for *InR‑intron5* (Figure 7G–L).

### 2.8 Integrated Model of IgLec Gene Family Organization and Antiviral Regulatory Network

**FIGURE 8:**
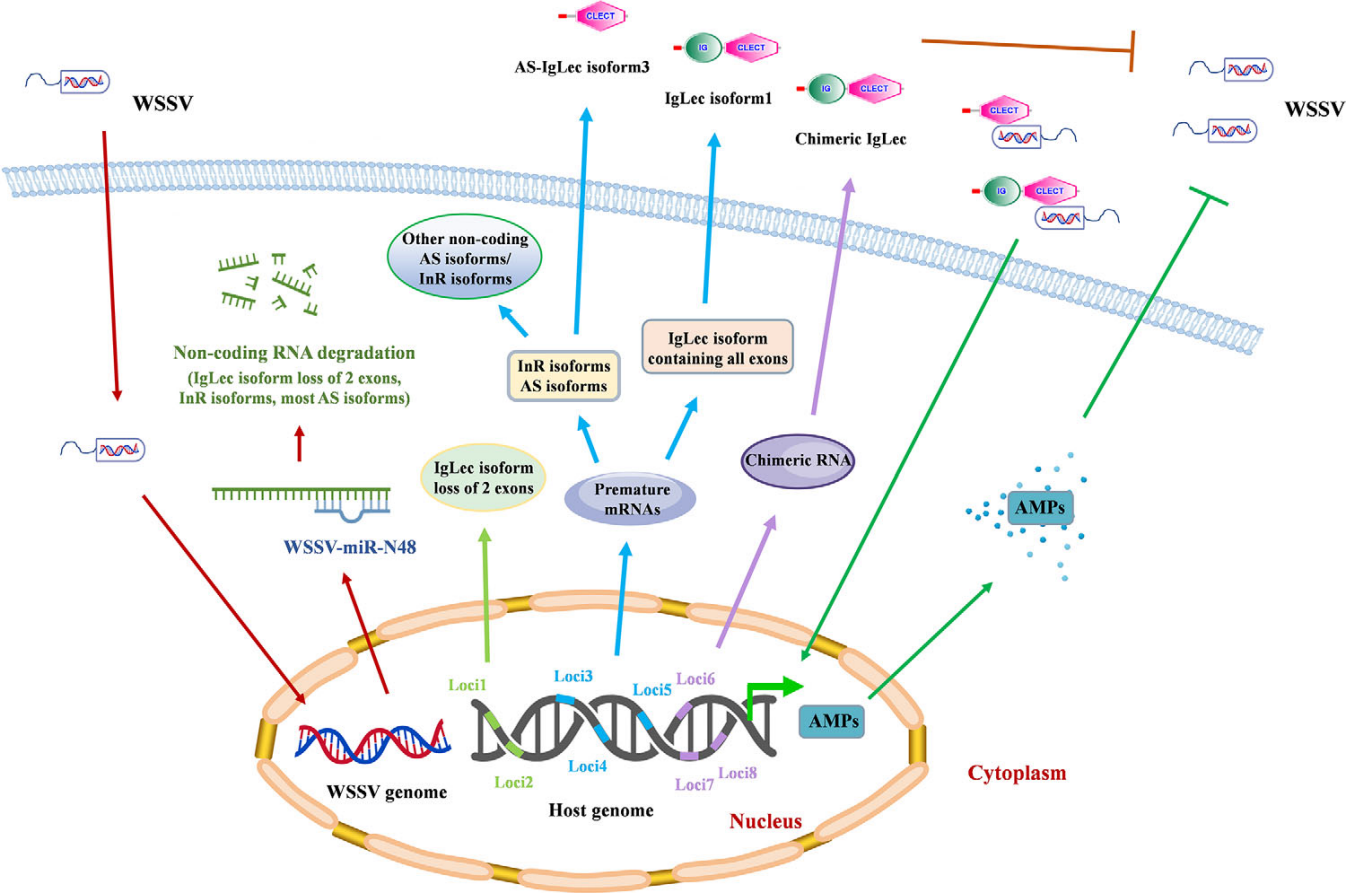
Schematic model of *IgLec* gene family organization and regulatory mechanism. The *IgLec* family employs multi‐locus duplication, alternative splicing, and inter‐locus recombination to generate diverse coding and non‐coding transcripts. Protein‐coding isoforms (IgLec isoform1 and AS‐IgLec isoform3) directly combat WSSV by modulating *AMP* expression. Non‐coding transcripts (exemplified by *InR‐intron3* and *InR‐intron5*) are preferentially targeted by viral miR‐N48, thereby buffering protein‐coding isoforms from repression. Depletion of these decoy transcripts renders protein‐coding isoforms vulnerable, compromising antiviral immunity.

The findings presented above are synthesized in a schematic model (Figure 8). The *IgLec* gene family employs three distinct mechanisms, multi‐locus duplication, alternative splicing, and inter‐locus genomic recombination, to generate diverse coding and non‐coding transcripts from five homologous genomic loci. Functional partitioning exists among transcript classes. Protein‑coding isoforms (IgLec isoform1 and AS‑IgLec isoform3) directly combat WSSV infection by binding to VP28 through their CRD, thereby modulating *AMP* expression. In contrast, non‐coding transcripts (exemplified by *InR‐intron3* and *InR‐intron5*) serve a regulatory function. Upon WSSV infection, viral‐encoded miR‐N48 preferentially targets and consumes these non‐coding decoy transcripts, thereby protecting the protein‐coding isoforms from repression. When non‐coding transcripts are depleted, viral miRNA gains access to and silences the functional isoforms, compromising antiviral immunity. This multilayered regulatory architecture suggests how genomic complexity and non‐coding transcript networks may be integrated to achieve fine‐tuned antiviral defense in crustaceans.

## 3 Discussion

The lineage‑restricted distribution of *IgLec* to Brachyura and Astacidea suggests that this gene may have emerged or been selectively retained in response to lineage‑specific ecological challenges, potentially reflecting distinct pathogen pressures encountered by these infraorders compared to other decapod groups. The presence of a structurally related protein in *V. parahaemolyticus* raises the possibility of horizontal gene transfer from bacteria to crustaceans, suggesting a potential case of inter‐kingdom gene transfer that may have contributed to immune diversification. Guided by these evolutionary features, the present study investigated how this gene has been integrated into the host immune system. The findings reveal that the *IgLec* gene family has evolved a multilayered genomic architecture that produces both protein‐coding and non‐coding transcripts. This complexity is generated by three mechanisms: multi‑locus duplication, inter‑locus recombination, and extensive alternative splicing.

Gene duplication and recombination have long been recognized as primary drivers of evolutionary innovation in immune gene families [18, 19]. Arthropods lack adaptive immunity and thus rely on the diversification of innate immune gene families for pathogen recognition and defense [20]. The identification of five homologous *IgLec* genomic loci in *E. sinensis* extends this paradigm by revealing that duplication events can generate loci with distinct exon‐intron architectures that enable functional specialization. The full‐length loci *gDNA1‐3* retain the capacity to produce functional protein effectors, whereas the truncated loci *gDNA4‐5* generate non‐coding transcripts. The presence of multiple such loci may facilitate rapid transcriptional bursts upon pathogen challenge, analogous to the multi‐copy organization of histone and rRNA gene clusters [21, 22]. This division of labor suggests that multi‑locus architecture, rather than being redundant, represents a dual‑mechanism antiviral strategy, one set of loci dedicated to effector protein production, and another specialized for non‑coding decoy generation. Notably, the overall predominance of non‐coding transcripts under basal conditions, which arises from multiple mechanisms including truncated loci and alternative splicing, may reflect a cost‐spreading strategy: maintaining a reservoir of readily disposable decoy transcripts avoids the constitutive production of costly effector proteins, whereas the rapid infection‐induced shift toward protein‐coding isoforms ensures timely deployment of antiviral activity. Beyond simple duplication, inter‐locus recombination between *IgLec* and its paralog *IgLec1* was identified, with short direct repeats mediating these events through replication slippage [23]. Such recombination‐mediated diversification may represent a general strategy in arthropods for generating immune receptor diversity in the absence of somatic rearrangement [24]. In addition to these genomic diversification mechanisms, extensive alternative splicing further expands the transcript repertoire derived from a limited number of loci, generating multiple variants.

Consistent with the established role of C‐type lectins as PRRs, the protein‐coding isoforms characterized in this study exhibit direct antiviral activity. This functional observation raises the question of why a larger suite of non‐coding transcripts is maintained alongside these functional effectors. Non‐coding transcripts are generated from multiple sources within the *IgLec* family, including truncated loci, intron‐retention, and exon‐skipping variants. Among these, the abundance of intron‐retaining transcripts is notable [25], as it contrasts with the view that intron retention primarily serves transcript degradation or results from splicing errors [26, 27]. To address this, we focused on intron‐retaining transcripts as representative non‐coding transcripts, exploiting their unique sequence composition to achieve specific knockdown.

Functional characterization revealed that these transcripts are rapidly downregulated following WSSV challenge and that their knockdown compromises protein‐coding isoforms during infection, indicating a protective function that becomes critical under challenge conditions. This protection is mediated by WSSV‐encoded miR‐N48, which directly targets the *IgLec* 3’UTR. Depletion of these decoy transcripts prior to viral miRNA exposure resulted in greater suppression of protein‐coding isoforms, demonstrating that non‐coding transcripts quantitatively buffer the impact of viral miRNA on functional transcripts. These findings extend the concept of non‐coding transcripts as molecular decoys or “sponges” in competing endogenous RNA (ceRNA) networks [14, 28, 29] in two respects. First, intron‐retaining transcripts, a class of non‐coding transcripts often overlooked, function as effective decoy transcripts in an in vivo host‑pathogen context. Second, host non‑coding transcripts bind to viral miRNAs, thereby competitively protecting protein‑coding transcripts. This reveals a direct molecular antagonism between host transcripts and viral miRNAs, representing a new dimension of the host‑virus arms race [30, 31]. The production of non‐coding transcripts is also observed in other IgLec family members, raising the possibility that similar decoy functions may exist in these members; however, this remains to be functionally tested. When these protective decoy transcripts are depleted, the protein‐coding isoforms become vulnerable, leading to impaired *AMP* expression, enhanced viral replication, and increased mortality.

Collectively, these findings support a model in which genomic complexity generates a transcript repertoire containing both effector and regulatory components: protein‐coding isoforms provide direct antiviral activity, whereas non‐coding transcripts act as decoy transcripts that are preferentially targeted by viral miRNA, thereby protecting functional isoforms from suppression. This buffering architecture enables rapid responses to viral challenge while protecting functional transcript expression from viral miRNA‐mediated suppression. However, direct evidence for physical sequestration, such as differential association with Argonaute‐containing complexes, remains to be established, and the molecular basis for preferential targeting of non‐coding transcripts is not fully understood. Notably, the 3’UTR sequences are identical between protein‐coding and intron‐retaining non‐coding transcripts, ruling out differences in the number of miR‐N48 binding sites as a simple explanation. A more plausible basis may lie in their distinct translation status and RNA structure, because non‐coding transcripts are not translated, their 3’UTR is not occupied by ribosomes, and intron retention may further expose the miR‐N48 binding site by altering overall secondary structure. In contrast, protein‐coding mRNAs may have this site partially occluded by translating ribosomes or sequestered within stable structures, thereby reducing miRNA‐mediated repression. Subcellular localization or transcript abundance could also contribute, although these possibilities remain to be tested. Addressing these questions and assessing whether similar decoy mechanisms operate in other arthropod immune gene families will be important directions for future work.

More broadly, the decoy mechanism described here may not be restricted to crustaceans; many immune gene families in invertebrates and vertebrates also produce abundant non‐coding transcript variants whose functions remain largely unexplored [32]. If similar decoy functions operate in these systems, it would suggest that innate immune gene evolution is shaped not only by selection on protein effectors but also by the emergence of regulatory RNA layers that buffer effector expression against pathogen‐encoded suppressors. These findings therefore suggest that non‐coding transcripts may not be incidental byproducts of genomic complexity but could instead act as active participants in antiviral defense, offering new insights into host‐virus interactions and pointing to a regulatory network that may balance immune competence against viral evasion.

## 4 Materials and Methods

### 4.1 Experimental Animals and Virus

Adult Chinese mitten crabs (*E. sinensis*, 20–30 g) were obtained from a commercial farm in Nanjing, China. Crabs were acclimated in laboratory tanks with aerated freshwater at 25 ± 1°C for one week and fed daily with a commercial diet. Only healthy crabs at the intermolt stage were used. The WSSV inoculum was provided by Shandong University. Viral copy numbers were quantified by TaqMan qPCR before use, and the stock was stored at −80°C.

### 4.2 Tissue Collection

For tissue distribution analysis, nerve, gills, hemocytes, intestine, eyestalk, and hepatopancreas were dissected from healthy crabs. Hemocytes were collected from hemolymph drawn from the walking leg base using a sterile syringe pre‐filled with ice‐cold anticoagulant (1.47 g glucose, 0.48 g citric acid, 1.32 g trisodium citrate, and ddH_2_O to 100 mL, pH 7.3), then pelleted by centrifugation at 800 × *g* for 10 min at 4°C. For viral challenge experiments, crabs received intramuscular injection of 100 µL WSSV suspension (10^6^ copies/mL) or sterile phosphate‐buffered saline (PBS). Hepatopancreas samples from three crabs per group were collected at 0, 24, 36, and 48 hpi. All tissues were immediately frozen in liquid nitrogen and stored at −80°C.

### 4.3 Genomic DNA Isolation and IgLec Locus Amplification

Genomic DNA was extracted from hepatopancreas using the NucleoSpin Tissue Kit (Clontech, USA). *IgLec* genomic loci were amplified with gene‐specific primers (Table S1) using 2× Taq PCR Master Mix (Generay Biotech, China). Thermocycling conditions were 94°C for 3 min; 35 cycles of 94°C for 30 s, 55°C for 45 s, and 72°C for 5 min; followed by 72°C for 10 min. For regions not covered by initial PCR, genome walking was performed using the Universal GenomeWalker 2.0 Kit (Clontech, USA) with two rounds of nested gene‐specific primers designed based on known sequences (Table S1). All amplicons were sequenced (Springen, China).

### 4.4 Identification of Chimeric Genes and Splice Variants

To identify chimeric genes generated by inter‐locus recombination between *IgLec* and *IgLec1*, primers spanning predicted recombination sites (Table S1) were designed and used to amplify genomic DNA. For splice variant identification, total RNA was extracted from hepatopancreas using the RNAprep Pure Tissue Kit (TIANGEN, China). cDNA was synthesized from 1 µg total RNA using TransScript All‐in‐One First‐Strand cDNA Synthesis SuperMix with One‐Step gDNA Removal (TransGen Biotech, China). PCR was performed with primers spanning multiple exons (Table S1) using ExTaq Polymerase (TaKaRa, Japan). Both PCR amplifications were carried out under the following conditions: 94°C for 3 min; 30 cycles of 94°C for 30 s, 55°C for 45 s, and 72°C for 60 s; and 72°C for 5 min. PCR products were gel‐purified (Omega Bio‐Tek, USA), ligated into pMD19‐T vector (TaKaRa, Japan), transformed into *Escherichia coli* DH5α, and sequenced (Springen, China).

### 4.5 Bioinformatics Analysis

To identify *IgLec* homologs, BLASTP searches were performed against the NCBI non‐redundant protein database using the *E. sinensis IgLec* sequence as query, with putative orthologs defined by E‐value < 1e^−^^5^, sequence coverage > 50%, and reciprocal best BLAST hit. Additional tBLASTn searches were conducted against transcriptomic and genomic datasets from multiple decapod species. Protein domains were predicted using SMART and InterProScan. Phylogenetic analysis was conducted using *cox1* gene sequences from 20 species (Figure 1A). A neighbor‐joining tree was constructed in MEGA 11 with the Kimura 2‐parameter model and 1000 bootstrap replicates. Multiple sequence alignments of IG domains were generated with Clustal Omega. Conserved motifs were identified using MEME (maximum 10 motifs, width 21–50 amino acids). Tertiary structures were modeled with SWISS‐MODEL. Putative miRNA targets in the *IgLec* 3’UTR were predicted using TargetScan, miRanda, and PicTar.

### 4.6 RT‐qPCR

RT‐qPCR was performed using TB Green Premix Ex Taq II (Takara, Japan) on a LightCycler 96 System (Roche, Switzerland). Each 20 µL reaction contained 10 µL TB Green Premix, 0.4 µL each primer (10 µm), 2 µL cDNA, and 7.2 µL RNase‐free water. Thermal cycling: 95°C for 30 s; 45 cycles of 95°C for 10 s and 60°C for 30 s. Melting curve analysis verified primer specificity. All reactions were performed in triplicate. Relative expression was calculated using the 2^−ΔΔCt^ method with *β‐actin* as internal control. Primer sequences are listed in Table S1.

### 4.7 Semiquantitative RT‐PCR

cDNA was amplified by reverse transcription PCR (RT‐PCR) using 2× Taq Master Mix (Vazyme, China) with the following conditions: 94°C for 3 min; 30 cycles of 94°C for 30 s, 55°C for 30 s, and 72°C for 20 s; followed by 72°C for 5 min. Products were separated on 1.5% agarose gels stained with GelRed (Biotium, USA) and visualized under UV light. *β‐actin* was amplified as an internal control.

### 4.8 Recombinant Protein Expression and Purification

Coding sequences of *IgLec isoform1*, IG of *IgLec isoform1* (*IgLec‐IG*), and *AS‐IgLec isoform3* were cloned into pET‐32a vector (Novagen, USA) and transformed into *E. coli* BL21 (DE3). Protein expression was induced with 0.5 mm isopropyl β‐D‐1‐thiogalactopyranoside at 16°C for 16 h. Bacterial cells were harvested, resuspended in lysis buffer (50 mm NaH_2_PO_4_, 300 mm NaCl, 10 mm imidazole, pH 8.0), and disrupted by sonication. Recombinant proteins were purified using Ni‐NTA agarose (GE Healthcare, USA), analyzed by SDS‐PAGE, and quantified by BCA assay (Beyotime, China). Recombinant thioredoxin (rTRX) protein from empty pET‐32a was purified similarly as a negative control.

### 4.9 GST Pull‐Down Assay

GST‐tagged VP28 (rVP28‐GST) and GST alone (negative control) were immobilized on glutathione‐Sepharose 4B beads (GE Healthcare, USA) at 4°C for 2 h. Recombinant His‐tagged IgLec isoform1, IgLec‐IG, or AS‐IgLec isoform3 (500 µg each) were incubated with the beads in binding buffer (50 mm Tris‐HCl, pH 7.5, 150 mm NaCl, 1 mm dithiothreitol, 0.1% Nonidet P‐40, 5 mm CaCl_2_) at 4°C overnight with gentle rotation. Beads were washed five times with binding buffer, and bound proteins were eluted by boiling in SDS‐PAGE loading buffer. Eluted samples were separated by 12% SDS‑PAGE and subjected to Western blot analysis using anti‑His and anti‑GST antibody (1:5000, Abcam, UK) to detect the pulled‑down proteins.

### 4.10 3’ RACE and Plasmid Construction

The *IgLec* 3’UTR was obtained by 3’ RACE using the Advantage 2 PCR Kit (Clontech, USA) with gene‐specific primers (Table S1). For luciferase assays, the 3’UTR was cloned into pmirGLO vector (Promega, USA) via *Sac* I and *Xba* I sites, generating pmirGLO‐*IgLec*‐3’UTR‐wt. For EGFP‐based assays, the 3’UTR was inserted into pEGFP‐C3 vector (Invitrogen, USA) downstream of EGFP using *Sac* I and *BamH* I sites, yielding pEGFP‐C3‐*IgLec*‐3’UTR‐wt. A mutant version with the WSSV‐miR‐N48 seed sequence “CTCCTCG” altered to “TGATCTA” was generated using the Mut Express II Fast Mutagenesis Kit V2 (Vazyme, China), producing pmirGLO‐*IgLec*‐3’UTR‐mut and pEGFP‐C3‐*IgLec*‐3’UTR‐mut. All plasmids were verified by sequencing.

### 4.11 Cell Culture and Transfection

Human embryonic kidney 293T cells were cultured in Dulbecco’s Modified Eagle Medium with 10% fetal bovine serum at 37°C and 5% CO_2_. Cells were seeded into 24‐well plates and transfected at 70–80% confluence using Lipofectamine 3000 (Invitrogen, USA). For luciferase assays, cells were co‐transfected with reporter plasmids (100 ng/well) and WSSV‐miR‐N48 mimic (5’‐ACGAGGAGAUGGUUGGGGACU‐3’; 100 nm/well; GenePharma, China) or negative control mimic (NC; 5’‐AGUGAGACGGGAGUACUGUGG‐3’; 100 nm/well). Luciferase activity was measured at 12, 24, and 36 hpt using the Dual‐Luciferase Reporter Assay System (Beyotime, China). Firefly luciferase activity was normalized to *Renilla* luciferase activity. For EGFP assays, cells were co‐transfected with EGFP reporter constructs (200 ng/well) and WSSV‐miR‐N48 mimic or NC (100 nm/well). EGFP expression was visualized 24 hpt by fluorescence microscope (Carl Zeiss, Germany), and quantified using a FlexStation II microplate reader (Molecular Devices, USA) with excitation at 490 nm and emission at 510 nm.

### 4.12 FISH and Confocal Microscopy

Hemocytes were collected from crabs at 48 h post‐WSSV injection. Cells were fixed with 4% paraformaldehyde in PBS for 30 min at room temperature, washed twice with PBS, and then permeabilized with 0.5% Triton X‐100 for 10 min. For FISH, fixed hemocytes were hybridized with FAM‐labeled *IgLec* mRNA probe (green; 5’‐FAM‐CAGAGGCAGTGGCGGTAGTG‐3’) and a Cy3‑labeled LNA‑modified oligonucleotide probe for WSSV‑miR‑N48 (red; 5’‐Cy3‐AGTCCCCAACCATCTCCTCGT‐3’) at 37°C overnight in hybridization buffer (40% formamide, 2× saline‐sodium citrate (SSC), 10% dextran sulfate, 1× Denhardt's solution). A scrambled CY3‑labeled LNA probe (5’‑CY3‑CCACAGTACTCCCGTCTCACT‑3’) was used as a negative control. After hybridization, cells were washed twice with 2× SSC containing 0.1% Tween‐20 at 37°C for 15 min, followed by one wash with 1× SSC under the same conditions. Nuclei were counterstained with 4’,6‐diamidino‐2‐phenylindole (DAPI; blue; 1 µg/mL; Beyotime, China) for 10 min at room temperature. Cells were mounted on glass slides with anti‑fade mounting medium (Beyotime, China) and observed under a confocal laser scanning microscope (Zeiss LSM 880, Germany).

### 4.13 SiRNA Knockdown Experiments

The siRNAs targeting transcripts retaining intron 3 (*InR‐intron3*) or intron 5 (*InR‐intron5*) were synthesized by GenePharma (China). The sequences were *InR‐intron3‐*siRNA, 5’‐UACACGAGUGCUGGCUGCCUUGUAU‐3’; *InR‐intron3‐*siRNA‐NC, 5’‐GUAGUCUAGCGAGGUCCAUUUGCCU‐3’; *InR‐intron5‐*siRNA, 5’‐CGGCGACUCCCGCCGCCCACUGUUU‐3’; *InR‐intron5‐*siRNA‐NC, 5’‐GCGUCGCCUUGGCCCCUAGCUCACC‐3’. Crabs received intramuscular injection of 100 µL siRNA (20 µM) or siRNA‐NC. For WSSV challenge, crabs were injected with WSSV (10^6^ copies/mL) at 12 h post‐siRNA injection. Hepatopancreas samples were collected at 24 and 48 h for RT‐qPCR analysis.

### 4.14 WSSV‐miR‐N48 Agomir Injection

WSSV‐miR‐N48 agomir (cholesterol‐conjugated and 2’‐O‐methyl‐modified miRNA mimic) and negative control agomir were synthesized by GenePharma (China). Crabs received intramuscular injection of 100 µL agomir (20 µM) or control agomir. For combined treatments, crabs received siRNA injection followed by co‐injection of WSSV‐miR‐N48 agomir and WSSV at 12 h post‐siRNA injection. Hepatopancreas samples were collected at 24 and 48 h for RT‐qPCR analysis.

### 4.15 Western Blot Analysis

Protein samples from crab hepatopancreas or recombinant protein preparations were separated by 12% SDS‐PAGE and transferred to polyvinylidene fluoride membranes (Millipore, USA). Membranes were blocked with 5% skim milk in Tris‐buffered saline containing 0.1% Tween‐20 (TBST), incubated overnight at 4°C with primary antibodies (anti‐VP28, 1:2000; anti‐glyceraldehyde‐3‐phosphate dehydrogenase (GAPDH), 1:10000; anti‐His tag, 1:5000; anti‐GST tag, 1:5000, Abcam, UK), then incubated with horseradish peroxidase‐conjugated secondary antibodies (1:10000 dilution; Abcam, UK) for 1 h at room temperature. Protein bands were visualized using enhanced chemiluminescence substrate (Thermo Fisher Scientific, USA). GAPDH served as a loading control for tissue samples.

### 4.16 WSSV Genomic Copy Number Quantification

Viral DNA was extracted from hepatopancreas using the TIANamp Genomic DNA Kit (TianGen, China). WSSV copy numbers were quantified by TaqMan qPCR using WSSV‐specific primers and probe (Table S1). Each 25 µL reaction contained 12.5 µL Premix ExTaq (TaKaRa, Japan), 0.5 µL each primer (10 µm), 1 µL probe (10 µm), 1 µL DNA template, and 9.5 µL ddH_2_O. Cycling conditions were 95°C for 1 min; 40 cycles of 95°C for 30 s, 52°C for 30 s, and 72°C for 30 s. A standard curve was generated using serial dilutions (10^8^ to 10^1^ copies/µL) of a plasmid containing the WSSV target sequence.

### 4.17 Survival Analysis

For survival experiments, crabs (n = 30 per group) were treated as described and monitored daily for mortality over 8 days. Cumulative survival rates were calculated and plotted as Kaplan–Meier survival curves using GraphPad Prism. Statistical significance was determined by the log‐rank test.

### 4.18 Statistical Analysis

All data are presented as mean ± standard deviation (SD) from at least three independent biological replicates. Statistical analyses were performed using SPSS (version 22.0, IBM, USA). Comparisons between two groups were analyzed by Student's *t*‐test. Comparisons among multiple groups were analyzed by one‐way analysis of variance (ANOVA) followed by Tukey's post‐hoc test. Survival curves were analyzed using the log‐rank test. Statistical significance was set at ^*^ *p* < 0.05, ^**^ *p* < 0.01, and ^***^ *p* < 0.001. Different letters indicate significant differences at *p* < 0.05.

### Author Contributions

Y.H. and Q.R. conceptualized and designed the experiments; Y.H., R.S., X.H. and G.Q.J. conducted experiments; Y.H. and Q.R. contributed new reagents/analytic tools; Y.H., R.S., X.H., G.Q.J., J.F.L. and Q.R. analyzed data; Y.H. and Q.R. wrote the paper.

## Ethics Statement

The sampling protocol was approved by the Ethics Committee of Experimental Animals at Hohai University (Approval No. HHU‐2023‐016) and adhered to the Animal Care Guidelines issued by the Ministry of Science and Technology (China). No wild animals were used in this study.

## Conflicts of Interest

The authors declare no conflicts of interest.

## Supporting information




**Supporting File**: advs77981‐sup‐0001‐SuppMat.docx.

## Data Availability

All data in this study are available in the article or in Supporting Information.
