# Supplementary material for: Non‐Coding Transcripts From Diversified Members of IgLec Family Protect Antiviral Effectors From Viral miRNA
Source: Adv Sci (Weinh). 2026 Sep 27:e77981. Online ahead of print. doi: 10.1002/advs.77981 (PMC13616369; doi:10.1002/advs.77981)
Supplement: Supplementary file 1 [file ADVS-9999-e77981-s001.docx]

**Table S1**

Primer sequences used in this study.

| **Primers** | **Nucleotide Sequence (5’-3’)** |
| --- | --- |
| **Genome amplification**  *IgLec*-gF  *IgLec*-gR  **Gene cloning**  *IgLec*-F  *IgLec*-R  **RT-qPCR**  *IgLec isoform1*-qF  *IgLec isoform1*-qR  *AS-IgLec isoform3*-qF  *AS-IgLec isoform3*-qR  *InR-intron3*-qF  *InR-intron3*-qR  *InR-intron5*-qF  *InR-intron5*-qR  *ALF2*-qF  *ALF2*-qR  *ALF3*-qF  *ALF3*-qR  *ALF4*-qF  *ALF4*-qR  *Cru4*-qF  *Cru4*-qR  *WAP*-qF  *WAP*-qR  *Lyso3*-qF  *Lyso3*-qR  *Lyso4*-qF  *Lyso4*-qR  *VP28*-qF  *VP28*-qR  *β-actin*-qF  *β-actin*-qR  WSSV-qF  WSSV-qR  WSSV-probe  **Protein expression**  IgLec isoform1-exF  IgLec isoform1-exR  AS-IgLec isoform3-exF  AS-IgLec isoform3-exR  IgLec-IG-exF  IgLec-IG-exR  **3’UTR cloning and mutagenesis**  pmirGLO-*IgLec-*3’UTR-F  pmirGLO-*IgLec*-3’UTR-R  pmirGLO-*IgLec*-3’UTR-mutF  pmirGLO-*IgLec*-3’UTR-mutR  pEGFP-C3-*IgLec*-3’UTR-F  pEGFP-C3-*IgLec*-3’UTR-R  pEGFP-C3-*IgLec*-3’UTR-mutF  pEGFP-C3-*IgLec*-3’UTR-mutR | ATGTCTTGCGTTGCCCGCGTCCTCTT  CTACGAGACTTGCGCCAGCAGGTT  ATGTCTTGCGTTGCCCGCGTCCTCTT  CTACGAGACTTGCGCCAGCAGGTT  GACGCCAGAGCCTGCGAGT  CTTGCCATACGTCTCCAAG  GTCTTCCAGCCTGCGAGTC  GATCTTGCCATCAGTCCATT  TACTGACGAGGTGGGAGAAG  GTGTATACAGCGTTGACAGGAG  TGGAACCACATCTTGGAGAC  GCGTTAGTGACGACAAACAAA  GACCAGGGTGGTGAACAAA  AGCATTCAGCAAAGGGTCA  GTCTCTGGTCTATGGCACAAC  TTGCCCTCGTGGTACAATTC  GACTTAGGAGTGCTTGCCAT  CATGCCTGGAGTTCCACTT  CCTGGTTCAGTGTACTGTTGT  TGGATAGTAGGAGGGCAGAC  GACGTGTCCAGTCATTCAGTT  GACGAGGAAGCAGCACTTT  GGTCGCCTATGTCAACAAGT  ACTCGTCCTTCACCCAGAT  CTACGTTTCAAGCGATGGATG  GTACCAGTACCAGTAGGTGATG  AGCTCCAACACCTCCTCCTTCA  TTACTCGGTCTCAGTGCCAGA  CGAGACCACTTACAACTCCATC  AGGGCAGTGATTTCCTTCTG  TTGGTTTCAGCCCGAGATT  CCTTGGTCAGCCCCTTGA  FAM-TGCTGCCGTCTCCAA-TAMRA  GCCATGGCTGATATCGGATCCACCTCCCTCGTCCCCGC  ACGGAGCTCGAATTCGGATCCCGAGACTTGCGCCAGCAGG  GCCATGGCTGATATCGGATCCACCTCCCTCGTCCCCGC  ACGGAGCTCGAATTCGGATCCCGAGACTTGCGCCAGCAGG  GCCATGGCTGATATCGGATCCTCCCCGGGCGTGCC  ACGGAGCTCGAATTCGGATCCGCTGACGACGTTCCTGACAGT  TCTAGTTGTTTAAACGAGCTCTAGCCTCGCCCAGACACACTCCTC  TGCCTGCAGGTCGACTCTAGAGCCAAGGTTATAGTGGTGATGGCG  AGCCTCGCCCAGACACATGATCTAGTCTTGCG  TGTCCTGGTTCGCAAGACTAGATCATGTGTCT  AAGTACTCAGATCTCGAGCTCTAGCCTCGCCCAGACACACTCCTC  TTATCTAGATCCGGTGGATCCGCCAAGGTTATAGTGGTGATGGCG  AGCCTCGCCCAGACACATGATCTAGTCTTGCG  TGTCCTGGTTCGCAAGACTAGATCATGTGTCT |
